# Effect of Titrated Exposure to Non-Traumatic Noise on Unvoiced Speech Recognition in Human Listeners with Normal Audiological Profiles

**DOI:** 10.1177/23312165221117081

**Published:** 2022-08-05

**Authors:** Mengchao Zhang, Richard M. Stern, Deborah Moncrieff, Catherine Palmer, Christopher A. Brown

**Affiliations:** 1 Audiology Department, School of Life and Health Sciences, 1722 Aston University, Birmingham, B4 7ET, UK; 2 Department of Electrical and Computer Engineering, 6612 Carnegie Mellon University, Pittsburgh, Pennsylvania 15213, USA; 3 School of Communication Sciences and Disorders, 5415 University of Memphis, Memphis, Tennessee 38152, USA; 4 Department of Communication Science and Disorders, University of Pittsburgh, Pittsburgh, Pennsylvania 15260, USA

**Keywords:** noise exposure, spectrotemporal envelope processing, speech in noise, cochlear synaptopathy

## Abstract

Non-traumatic noise exposure has been shown in animal models to impact the processing of envelope cues. However, evidence in human studies has been conflicting, possibly because the measures have not been specifically parameterized based on listeners’ exposure profiles. The current study examined young dental-school students, whose exposure to high-frequency non-traumatic dental-drill noise during their course of study is systematic and precisely quantifiable. Twenty-five dental students and twenty-seven non-dental participants were recruited. The listeners were asked to recognize unvoiced sentences that were processed to contain only envelope cues useful for recognition and have been filtered to frequency regions inside or outside the dental noise spectrum. The sentences were presented either in quiet or in one of the noise maskers, including a steady-state noise, a 16-Hz or 32-Hz temporally modulated noise, or a spectrally modulated noise. The dental students showed no difference from the control group in demographic information, audiological screening outcomes, extended high-frequency thresholds, or unvoiced speech in quiet, but consistently performed more poorly for unvoiced speech recognition in modulated noise. The group difference in noise depended on the filtering conditions. The dental group's degraded performances were observed in temporally modulated noise for high-pass filtered condition only and in spectrally modulated noise for low-pass filtered condition only. The current findings provide the most direct evidence to date of a link between non-traumatic noise exposure and supra-threshold envelope processing issues in human listeners despite the normal audiological profiles.

## Introduction

For many decades, noise exposure has been assumed to be safe or ‘non-traumatic’ if the noise does not lead to permanent threshold shifts (Saunders et al., 1985; Eggermont, 2017). However, accumulating evidence over the past ten years indicates that non-traumatic noise exposure could lead to auditory pathophysiological changes that are undetected by routine audiological exams (Kujawa & Liberman, 2009; Lin et al., 2011; Furman et al., 2013; Valero et al., 2017; Munguia et al., 2013; Sheppard et al., 2017; Pienkowski & Eggermont, 2009; Pienkowski & Eggermont, 2010a, 2010b; Pienkowski et al., 2011; Zhou & Merzenich, 2012; Fernandez et al., 2020). These physiological changes could occur at the auditory-nerve level, such as damage to the synapses of a selective group of auditory nerve fibers responsible for encoding high-intensity sound (termed ‘cochlear synaptopathy’; Kujawa & Liberman, 2009; Lin et al., 2011; Furman et al., 2013), and/or occur along the central auditory pathway, such as increase spontaneous activity (Pienkowski & Eggermont, 2009; Munguia et al., 2013; Pienkowski et al., 2013), reduced number of inhibitory neurons (Zhou & Merzenich, 2012; Munguia et al., 2013; Kamal et al., 2013; Lau et al., 2015), broadened frequency tuning curves (Zhou et al., 2011; Zhou & Merzenich, 2012; Kamal et al., 2013), reduced adaption of firing rates to sound level statistics (Bakay et al., 2018), and disrupted cortical tonotopic representation (Pienkowski & Eggermont, 2009, 2010a, 2010b; Pienkowski et al., 2011, 2013).

The current study examines one of the plausible perceptual effects of non-traumatic noise exposure, degraded supra-threshold envelope processing, which is thought to be a consequence of cochlear synaptopathy (Bharadwaj et al., 2014; Shaheen et al., 2015) though the contribution from the central auditory system is not ruled out. There has been a great deal of debates on the relationship between noise exposure and envelope processing as well as other types of auditory processing in human listeners, because the evidence has been rather inconsistent (Kumar et al., 2012; Stone et al., 2008; Stone & Moore, 2014; Paul et al., 2017, 2018; Prendergast et al., 2017a, 2017b; Yeend et al., 2017; Füllgrabe et al., 2020). It may be that human auditory perception is less susceptible to the noise exposure that does not cause permanent threshold shifts, or that researchers have overlooked consequential details in the measures or the participants. For instance, conventional tasks assessing temporal envelope processing like amplitude modulation (AM) detection or discrimination (e.g. Kumar et al., 2012; Stone et al., 2008; Paul et al., 2017) may not be always suitable for the purpose of this research, as the listeners may employ off-frequency cues for high-intensity presentation levels. Additionally, it is possible that behavioral differences will not be observed between those who have been excessively exposed and those who have not, if the differences of their exposure dosages have not reached some critical value.

The purpose of the current study is to examine the relationship between noise exposure and envelope processing abilities in humans using a different paradigm. To facilitate the distinctiveness of noise exposure profiles between the groups, dental school students were chosen as the experimental group and their non-dental-school peers as the control group. Most of the dental-school students are young adults who are less likely to have age-related hearing loss. Dental students receive regimented, well-defined exposure to dental drill noise with acoustic energy above 4 kHz (Fernandes et al., 2006; Choosong et al., 2011) throughout their program of study. As people in the general population typically get exposed to environmental noise that is often low-pass filtered below 2 kHz (Can et al., 2010; Bořil et al., 2012; Ramo et al., 2013; Albert & Decato, 2017), the noise exposure profiles of a group of non-dental young adults should show distinct difference from those of the dental young adults. Furthermore, the levels of the noise produced by modern-day dental devices do not exceed 85 dB SPL (Chen et al., 2013; Ai et al., 2017; da Cunha et al., 2017; Ahmed & Ali, 2017; Fernandes et al., 2006; Yousuf et al., 2014; Goswami et al., 2017) so the 8-h daily time-weight average (TWA) level of dental students range between 70 and 80 dB SPL (Choosong et al., 2011; Burk & Neitzel, 2016) below legislative standards (85 dBA for 8 h, NIOSH, 1998). Taken together, these factors make dental school students a viable population to study non-traumatic noise exposure.

The current study uses recognition of unvoiced speech as a measure to assess envelope processing skills. Speech can be decomposed into temporal envelopes and temporal fine structures (TFS) (Rosen, 1992; Moore, 2019). When the TFS is replaced by random noise, speech becomes unvoiced (Kawahara & Irino, 2005; Kawahara et al., 2009) and speech intelligibility depends solely on the spectro-temporal modulations (i.e., envelopes) of the original speech. Like natural speech, intelligibility of unvoiced speech avoids potential interference by off-frequency listening because each frequency region contributes to speech differently. Despite the removal of TFS, unvoiced speech can still be highly redundant both acoustically and linguistically (Shannon et al., 1995; Loizou et al., 1999; Tillery et al., 2012; Brown, 2014, 2018). Therefore, acoustic redundancy of the unvoiced speech was controlled by filtering so the stimulus spectra fell inside or outside the spectrum of dental noise. Linguistic redundancy of the speech was constrained using speech materials with low contextual cues. Lastly, given that behavioral studies have primarily focused on temporal aspects of envelope processing (e.g. Bharadwaj et al., 2014), it is of interest whether noise exposure impacts spectral aspects of envelope processing as well. By adding temporally or spectrally modulated noise, the study examined the exposure impact on temporal or spectral envelope processing, respectively.

Routine audiological screening results and the recognition of unvoiced speech in quiet were also assessed to examine whether (1) the experimental and control groups will perform differently on unvoiced speech perception in quiet, and (2) whether non-traumatic noise exposure is related to supra-threshold envelope processing in the absence of audiologically relevant peripheral changes. In theory, because non-traumatic noise exposure is thought to impact supra-threshold auditory perception, it should not impact speech perception in quiet and audiological screening outcomes. We tested these hypotheses by examining the two groups on measures of pure tone hearing sensitivity, acoustic reflexes, otoacoustic emissions, and unvoiced speech recognition in quiet.

## Methods

### Participant Screenings

Participants were recruited through recruitment ads, emails, and the Pitt  +  Me participant recruitment service sponsored by the University of Pittsburgh. All research protocols were approved by the Human Research Protection Office at the University of Pittsburgh and all participants provided written consent of participation. Before attending the formal test, participants passed demographic and audiological screenings, and a task familiarization session.

#### Demographic Screening

Participants filled out a questionnaire regarding their demographic information and noise exposure history. Specifically, eligible participants met the following criteria: 1) between 22 and 30 years of age; 2) speaking English as the first and the only language; 3) no known hearing issues in the past or at the time of screening, including otologic disorders, ear infection, otitis media, or hearing loss; 4) no neurological, neurophysiological or neuropsychological disorders, and no brain trauma; 5) perception of tinnitus allowed for the experimental group only if it occurred since starting dental school; 6) no history of frequent exposure to impulsive noise; 7) occupational noise exposure cannot exceed NIOSH standard (85 dBA for 8 h, NIOSH, 1998).

Participants’ lifetime noise exposure dose was determined by a questionnaire that surveys the frequencies and the sound levels of noisy activities. The Exposed Noise and Hearing Disorders of Conscripts (ENHDC) questionnaire was chosen as it includes the wide range of noisy activities and allows the calculation of lifetime noise exposure dose in unit of dB SPL, informing whether the participant's past exposure has been non-traumatic. The ENHDC was first developed by Jokitulppo et al. (2006) and was revised to fit the goals of this study to include more noisy activities and collect the exposure schedule over a finer time scale, including the past 1-year, 3-year, as well as the rest of the participants’ life. The activities reported are listed in Table 1. For Parts 1 to 3, all participants reported on: (1) the estimated loudness from 1 to 5 which was based on the criteria described in Jokitulppo et al., (2006), (2) the number of hours per week participating in the activity, (3) the number of weeks of participation in the past 1 year (52 weeks), (4) the number of weeks of participation from 3 years ago up to 1 year ago (104 weeks), (5) the number of years of participation and number of weeks of participation per year from birth up to 3 years ago, and (6) percentage of time using hearing protection devices. Additionally, the dental students completed Part 4 on the use of each dental drill device for each of the four school years, including (1) the number of hours per week using the device, (2) the number of weeks using the device, and (3) percentage of time using hearing protection devices. The sound levels of the dental devices were measured by the first author at the dental school. The dental devices were operated by a 3^rd^-year dental student and the levels were measured 6 inches away from the operating device using a Larson Davis 824 Sound Level Meter (Larson Davis Inc, Depew, NY). Three measurements were performed for each device and the average levels were used in Part 4 of the noise exposure survey. The outcomes of the recording are provided in Table 1.

**Table 1.** Noisy Activities in the Noise Exposure Survey.

Sections and activities in the noise exposure survey
Part 1 – Leisure time noise
Watching TV Playing video/computer games Working out to music (when not working out) Listening to music, radio programs, etc. using personal headsets or earphones (when not working out) Listening to music, radio programs, etc. from audio speakers in a car or at home Watching movies in a theatre Going to bars or pubs Attending concerts and festivals with acoustic system (e.g. classical music) Attending concerts, events and festivals with amplified system (e.g. rock, pop, rally) Attending motor sports or ride/operate motorized vehicles such as motorcycles, jet skis, speed boats, snowmobiles, or four-wheelers Using tools indoors during unpaid time Using tools outdoors during unpaid time Attending or participating in indoor commercial/high-school sports events (e.g. ice-hockey, basketball) Attending or participating in outdoor commercial/high-school sports events (e.g. football, baseball) Attend car/truck races Ride in or pilot small aircraft/private airplanes
Part 2 Leisure time noise
Playing in a band or orchestra or singing in choir Practicing a musical instrument or vocal
Part 3 Occupational noise (non-dentistry noise)
Any work involving power tools, chainsaws, or other shop tools Any work using drive heavy equipment or loud machinery (such as tractors, trucks, or farming or lawn equipment like mowers/leaf blowers)
Part 4 Dental noise (completed only by the dental students)
Student handpieces High-speed turbine, 82.3 dB (A) Contra-angle handpiece, 70.3 dB (A) Straight handpiece, 69.3 dB (A) Clinic handpieces Ultrasonic scaler, 73.6 dB (A) High-speed turbine, 80.7 dB (A) Contra-angle handpiece, 62.3 dB (A) Straight handpiece, 62 dB (A) Lab-and-clinic equipment Polishing equipment, 82 dB (A) Vibrating equipment, 88 dB (A) Lathe equipment 3000, 93 dB (A) Stone trimmer, 82.3 dB (A) Low-volume suction pump, 68.3 dB (A) High-volume suction pump, 69.8 dB (A) Air-water syringe 60.7 dB (A) Sandblaster, 90 dB (A)

With the schedules and the levels available for each activity throughout a person's life, lifetime equivalent sound exposure level (L_eq_) can be calculated. Expressing L_eq_ in dB SPL allows us to make straightforward decisions about whether a participant's noise exposure has been non-traumatic.

EQ. 1 was used to calculate the exposure dose of noisy activities (NIOSH, 1998; Neitzel et al., 1999):

(EQ. 1) $$D_{i}=\frac{C_{i}}{(8760*N_{i})/2^{(L_{i}-79)/3}}\times 100$$

Where *N* is the number of years out of which the noise exposure needs to be computed, *C* is the number of hours participating in that activity during the time specified by number of years (i.e., *N*), *L* is the average sound pressure level of that activity, and *i* represents the ordinal number of each noisy activity. The number of hours participating in regular, less noisy activities was calculated by subtracting the total hours of noisy activity from the total hours of a participant's lifetime. These activities were assumed to occur at 64 dB SPL on average (Johnson et al., 2017). Likewise, the dose for regular activity was computed using EQ. 1. For dental-school participants, the total hours and the exposure dose of regular activity were re-calculated by subtracting the total hours of dental and non-dental noisy activities from total hours of the individual's lifetime.

The doses of all activities were then summed and used to compute L_eq_ using EQ. 2:

(EQ. 2) $$L_{eq}=\left[10\times log_{10}\left(\frac{\sum D_{i}}{100}\right)\right]+79$$

#### Audiological Screening

Participants received standard audiological assessments (Table 2) together with distortion product otoacoustic emissions (DPOAEs) and pure-tone audiometry at extended-high frequencies (EHFs, > 8 kHz) which may be more sensitive to noise exposure (Liberman et al., 2016). The audiologic screening included otoscopic exam, tympanometry, acoustic reflex, DPOAE, and pure-tone audiogram. The otoscopic exam was conducted at both ears using a handheld Welch Allyn otoscopy. Tympanogram was tested at both ears with a 226-Hz tone presented through a testing probe from GSI Tympstar Middle Ear Analyzer (Grason-Stadler Inc., Milford, NH).

**Table 2.** Exams, Devices, and Passing Criteria of the Audiological Screening.

Exams	Devices	Passing criteria
Otoscopic exam, both ears	Handheld Welch Allyn otoscopy	No occlusion, intact ear drum
Tympanometry to 226-Hz tone, both ears	GSI Tympstar Middle Ear Analyzer	- Compliance between 0.3 ml to 1.8 ml - Middle ear pressure between −150 daPa to + 150 daPa - Ear canal volume between 0.6 cc to 2.0 cc
Ipsilateral and contralateral acoustic reflexes to probe tones of 0.5, 1, 2, and 4 kHz presented at 95 dB SPL, both ears	GSI Tympstar Middle Ear Analyzer	Reflex response ≥ 0.02 ml for ipsilateral stimulation at left ear
DPOAE at f2 = 552, 698, 879, 1104, 1392, 1753, 2207, 2783, 3506, 4419, 5566, 7012, 8838, 11133, 14028, 17671 Hz, f2/f1 = 1.22, L1 = 65 dB SPL and L2 = 55 dB SPL, both ears	IHS	SNR ≥ 6 dB for 80% of the test points between 1 and 8 kHz
Pure-tone audiogram at 0.25, 0.5, 1, 2, 3, 4, 6, 8, 12.5, 14, and 16 kHz, both ears	Madsen Astera 2 with Otosuite™	Thresholds ≤ 20 dB HL from 0.25 to 8 kHz (ANSI, 2004)

Acoustic reflex was also conducted using GSI Tympstar Middle Ear Analyzer. Ipsilateral and contralateral acoustic reflexes were measured at each ear with a probe tone of 0.5, 1, 2, or 4 kHz presented at 95 dB SPL. The responses (in ml) of ipsilateral stimulation at the left ear were used to determine eligibility (Table 2) because the stimuli in the unvoiced speech tests were only presented at the left ear.

DPOAE was measured through Intelligent Hearing System (IHS, Miami, FL). The frequency range of the DPOAE spanned from 0.5 to 20 kHz with 3 frequency points per octave. The F2/F1 ratio was 1.22. The presentation levels for L1 and L2 were 65 and 55 dB SPL, respectively. Eligibility was determined based on the SNRs from 1 to 8 kHz (Table 2), and the SNRs from 8 to 16 kHz were used to analyze the effect of dental noise exposure on high-frequency hearing.

For the audiometric screening, pure tones ranged from 0.25 to 8 kHz (Table 2). A Madsen Astera 2 Audiometer (GN Otometrics, Denmark) controlled though the Otosuite^TM^ software was used to present the tones over a pair of ER-3 insert earphones to the participants. The participants sat inside a soundproof booth and pressed a handheld bottom to indicate response to the tone. Absolute thresholds were not searched for the screening frequencies. Participants who were able to hear at or below 20 dB HL at each frequency were considered eligible. Additionally, absolute thresholds were searched and recorded for the EHF tones at 12.5, 14, and 16 kHz at each ear with Sennheiser HD 800 headphones, and the thresholds at the EHFs were further analyzed. At each frequency, a tone was presented at 25 or 30 dB HL, decreased by 10 dB if it was heard, or increase by 5 dB if no response was given. The absolute threshold was defined as the lowest level where participants gave 2 out of 3 correct responses.

### Participant Information

Due to lack of previous reports using unvoiced speech recognition, a pilot study (EXP group, n  =  9; CTL group, n  =  7) was conducted, showing that effect size ranged from medium-large (Cohen's f  =  0.37) to large (Cohen's f  =  0.61) (Cohen, 1988). The total sample size for analysis of variance (ANOVA) for main effects and interactions was then estimated using G*Power 3.1 (*α*  =  0.05, 1- *β*  =  0.8) based on the pilot data, yielding a total sample size between 29 to 74 participants. Fifty-two of the originally recruited eighty-six participants (EXP group, n  =  25; CTL group, n  =  27) passed the demographic and audiological screenings and completed the experimental speech recognition tasks. Table 3 shows means and standard deviations of age, the number of years of musical training, and lifetime non-dental noise exposure L_eq_, lifetime dental noise exposure L_eq,_ and lifetime all noise exposure combined L_eq_ for both groups. A one-way ANOVA was conducted for each outcome variable. The lifetime L_eq_ with dental and non-dental noise combined was significantly higher for the EXP group than for the CTL group by about 3.6 dB, *F*(1, 50)  =  8.240, *p*  =  0.006. When considering the lifetime non-dental noise exposure L_eq_, there was no significant difference between the two groups, *p* > 0.05. There was also no significant difference between the two groups in age or in the years of musical training, *p* > 0.05.

**Table 3.** Demographics of the EXP and the CTL Groups.

	EXP group	CTL group	F statistics
N	25 (female, n = 15)	27 (female, n = 25)	
Population	2nd to 4th year dental students (3rd to 4th year n = 20)	Non-dental graduate students or professionals with at least bachelor's degrees	
Age (years)	25.3 ± 1.7	24.6 ± 2.1	1.847
Lifetime non-dental noise exposure L_eq_ (dB SPL)	75.3 ± 4.3	75.1 ± 5.6	0.028
Lifetime dental noise exposure L_eq_ (dB SPL)	74.6 ± 3.4	0	13164.561***
Lifetime all noise exposure combined L_eq_ (dB SPL)	78.7 ± 2.7	75.1 ± 5.6	8.240**
Music training (years)	2.7 ± 3.8	3.4 ± 4.7	0.292

Note. *, *p* < 0.05; **, *p* < 0.01; ***, *p* < 0.001.

For the EXP group, the lifetime dental noise exposure L_eq_ systematically and significantly increased with the number of years at dental school, where the L_eq_ of the 2^nd^, the 3^rd^, and the 4^th^ year students were 71.2 dB SPL (SD  =  1.4), 74.4 dB SPL (SD  =  3.2), 77.1 dB SPL (SD  =  2.6), respectively. Only 3 of 25 participants in the EXP reported the use of earplugs when they were using the student handpieces. Three different participants in the EXP group reported experience with ringing in the ear, with the tinnitus occurring either intermittently, randomly or at night.

### Stimuli

Target stimuli were IEEE sentences (IEEE, 1969) that were spoken by an adult female in standard English (sampling frequency 44.1 kHz, bandwidth 0.08 to 12 kHz). An unvoiced version of each token was produced by the TANDEM-STRAIGHT vocoder (Kawahara & Irino, 2005; Kawahara et al., 2009). The vocoder extracts the envelopes of the natural utterance and excites the envelopes with random noise, producing an unvoiced token with high spectral resolution (Kawahara et al., 2009). The unvoiced stimuli were low-pass (LPF) or high-pass filtered (HPF) using a 40th-order Butterworth infinite impulse response (IIR) filter. Based on pilot data, the cut-off frequencies of the narrowest bandwidth to achieve 90% intelligibility used cutoff frequencies of 2.3 kHz for the LPF condition and 1.7 kHz for the HPF condition.

**Figure 1.**
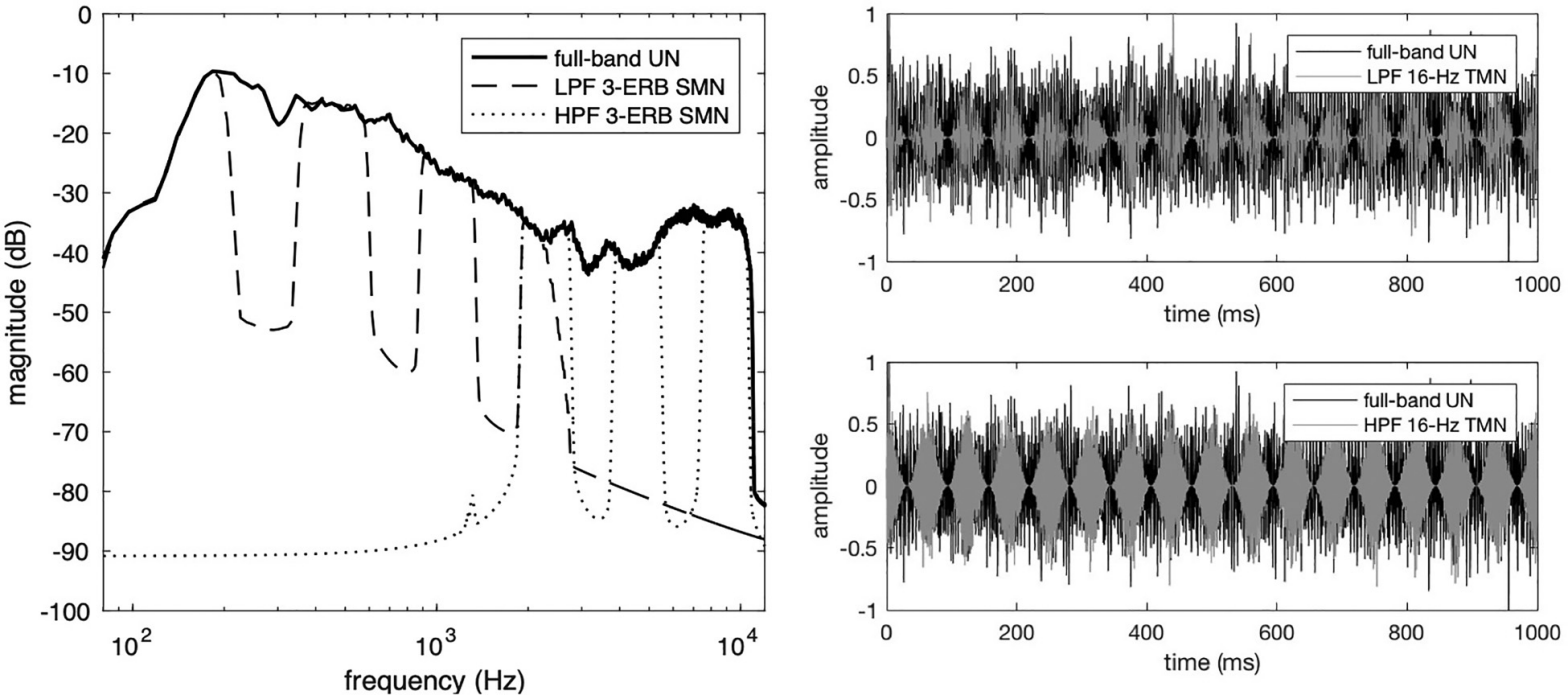
Left panel: spectra of the full-band unmodulated noise (solid), LPF SMN (dashed), HPF SMN (dotted). Right panel: time-domain waveforms of the full-band unmodulated noise (black), 16-Hz LPF TMN (top, grey), 16-Hz HPF TMN (bottom, grey).

Four different maskers were used, all of which were derived from Gaussian white noise that was spectrally shaped to match the long-term average spectrum of the IEEE sentences (Figure 1, left panel). The maskers were either an unmodulated noise (UN), one of two temporally modulated noises (TMN) (Figure 1, right panel), or a spectrally modulated noise (SMN). The parameters of the temporal and spectral gaps were determined by a pilot study to produce significant masking release. The TMNs were produced by sinusoidally amplitude-modulating the UN at 16 Hz or 32 Hz with a modulation depth of 1. The SMN contained spectral gaps that were 3 equivalent rectangular bands (ERB) wide and interleaved with passbands which were also 3 ERBs wide. The SMNs were processed by passing the UN through a bank of 40^th^-order Butterworth IIR band-pass filters. The frequencies of the unfiltered energy in the SMNs are listed in Table 4. All maskers were LPF or HPF in the same manner as the targets. The filtered maskers were then equated to the filtered targets in root-mean-square (RMS) levels.

**Table 4.** SMN Bands with Energy.

Overall filtering	Bands with energy (Hz)
Low pass	80–198
	360–585
	894–1322
	1913–2729
High pass	1913–2729
	3856–5413
	7562–10530

### Procedure

All psychophysical procedures were conducted in a soundproof booth. Stimulus presentation was controlled through MATLAB scripts on a MacBook Pro and presented monaurally to the left ear through a pair of AKG K240 MKII supra-aural headphones. The exposure is assumed to have equal effect on both sides, so testing either ear should not make a difference. However, binaural presentation activates the contralateral efferent suppression on the auditory nerves (Lisowska et al., 2008). Hence, monaural presentation was used to exclude the contribution from contralateral efferent system. Before formal testing, participants performed a familiarization task in which they were instructed to repeat forty filtered unvoiced sentences (20 for each filtering condition) in quiet with feedback. Those who scored less than 90% were given an additional 10 sentences in that filtering condition. Participants who could not score 80% were excluded from the study. All participants in the current study have scored 80% or more.

The first task was to recognize unvoiced speech in quiet. The lowest sound pressure level to achieve 50% correct responses (i.e., absolute speech recognition threshold [ASRT]) was measured through a one-down-one-up adaptive procedure (Levitt, 1971) which tracks the point for 50% correct responses on the psychometric function. The initial presentation level was 0 dB SPL where participants cannot perceive the target sentence. The level was then elevated if the participant gave an incorrect response or was reduced if the participant gave a correct response. The first sentence was repeated until the participant gave a correct response, and the rest of the sentences were presented only once. The step size was 4 dB initially and 2 dB after two reversals. A correct response required correctly identifying three or more key words. The omission of the ending ‘s’ was counted correct but the omission of ‘ed’ to indicate past tense or any phoneme substitution was scored as incorrect. Three IEEE lists (i.e., 30 sentences) were used for each filtering condition and the order of the filtering conditions was randomized. The measurement for a condition was stopped if the participant reached 10 reversals or completed 30 sentences, whichever came first. The ASRT was the average sound pressure levels at all but the first 2 reversals. No feedback was provided.

**Figure 2.**
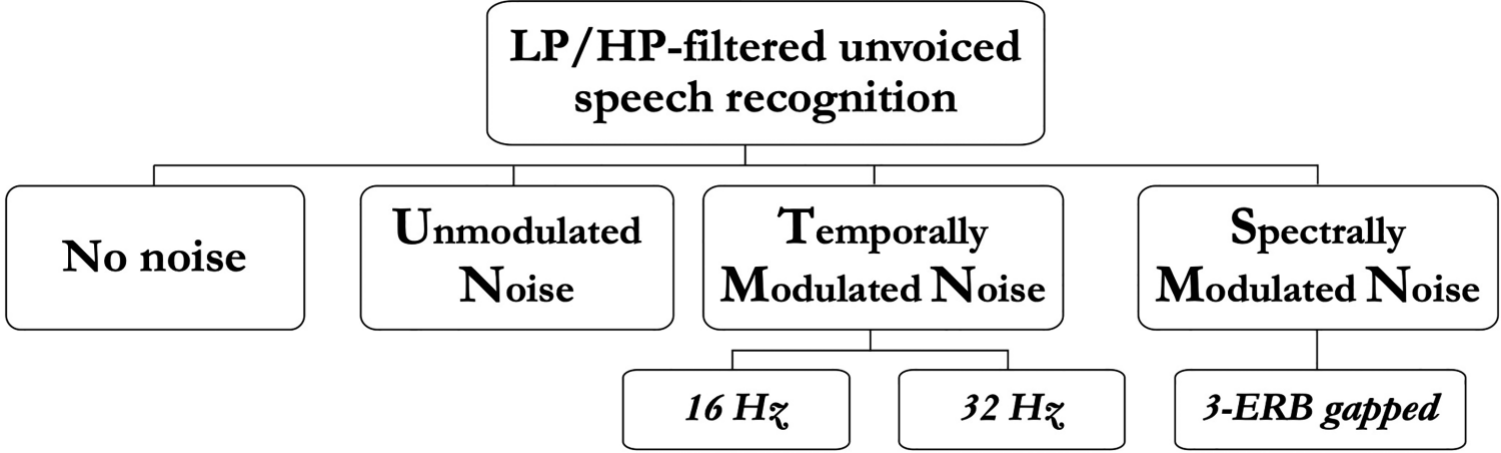
Tested conditions for unvoiced speech recognition task.

The second task was to recognize unvoiced speech in noise. The SNR for 50% correct responses (i.e., speech recognition threshold [SRT]) was measured through a one-down-one-up adaptive procedure. The noise level was fixed at 65 dB SPL and the target sentence level was adaptively varied. The first sentence was presented at −4 dB SNR and was repeated until the participant gave a correct response. The rules of scoring, step-size changing, and condition stopping were identical to those used in the speech in quiet task. Eight conditions (2 filtering conditions  ×  4 noise maskers) were tested (Figure 2) and the order of the conditions was randomized. Three IEEE lists were used for each condition and the SRT was the average SNR of all but the first 2 reversals.

All the data were analyzed in IBM SPSS® Statistics 26.0 and plotted in MATLAB.

## Results

First, audiological screening outcomes and unvoiced speech recognition performance in quiet were compared between the two groups. Next, unvoiced speech recognition in noise was compared for temporally and spectrally modulated noises. Lastly, correlation and regression analyses were conducted to examine the contributions of demographic factors and audiological screening outcomes.

### Audiological Screenings and Unvoiced Speech Recognition in Quiet

The average amplitudes of the middle ear acoustic reflex for probe frequencies from 0.5 to 4 kHz were compared between the two groups through one-way analysis of variance (ANOVA). The result showed that the reflex amplitude of the CTL group (0.12 ± 0.08 ml) was on average larger than that of the EXP group (0.09 ± 0.05 ml) but the difference was not statistically significant, *F*(1,50)  =  2.266, *p*  =  0.139.

The DPOAE amplitudes were analyzed through a 2 (group)  ×  3 (frequency) mixed-model ANOVA. Low, middle, and high frequency responses of the DPOAE were the average emission amplitudes from 0.55 to 2.8 kHz, from 3.5 to 8.8 kHz, and from 11.1 to 14 kHz, respectively. There was a significant main effect of frequency after Greenhouse-Geisser correction, *F*(1.748, 87.380)  =  105.025, *p* < 0.001, *η_p_^2^*  =  0.677, but no significant main effect of group or interaction between group and frequency, *p* > 0.05. The simple-effect multiple comparisons did not show a statistically significant group difference at any given frequency condition, *p* > 0.05.

The average thresholds of pure-tone audiogram at 12.5, 14, and 16 kHz (i.e., EHF) were analyzed through a one-way ANOVA with Bonferroni correction. The thresholds of the CTL group (6.6 ± 10.9 dB HL) were slightly lower than that of the EXP group (9.2 ± 10.1 dB HL) but the difference was not statistically significant, *F*(1,50)  =  0.792, *p*  =  0.378.

**Figure 3.**
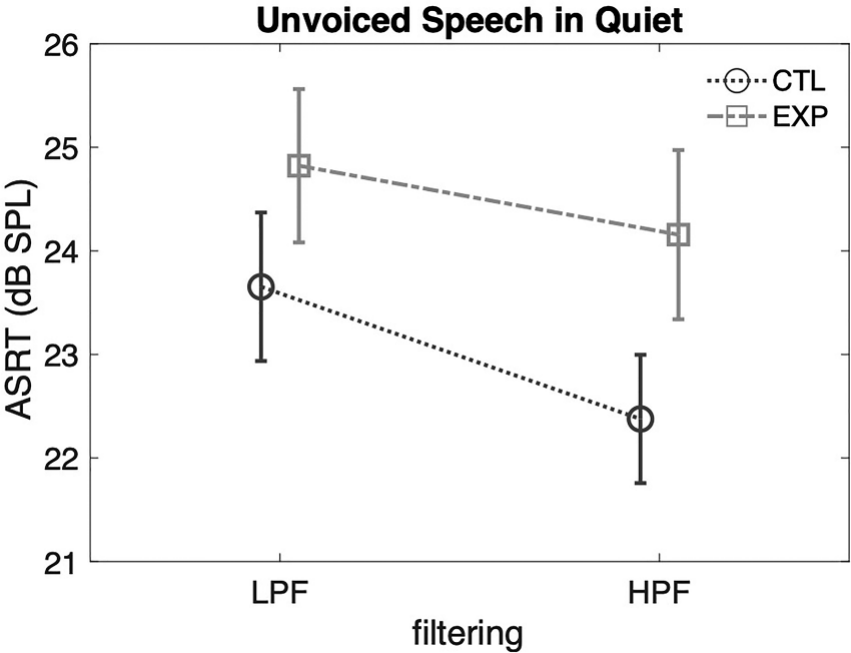
Performance of LPF and HPF unvoiced speech in quiet between the CTL (dark) and the EXP (light) groups. Error bars: the standard error of the mean (SEM).

The ASRTs for unvoiced speech recognition in quiet were analyzed through a 2 (group)  ×  2 (filtering) mixed-model ANOVA (Figure 3). There was no significant main effect of filtering, *F*(1, 50)  =  3.637, *p*  =  0.062, *η_p_^2^*  =  0.068, of group, *F*(1, 50)  =  2.757, *p*  =  0.103, *η_p_^2^*  =  0.052, or interaction between filtering and group, *F*(1)  =  0.360, *p*  =  0.551, *η_p_^2^*  =  0.007 (Figure 3). There was no significant difference in ASRT between the two groups for either filtering conditions, *p* > 0.05.

### Unvoiced Speech Recognition in Noise

The SRTs of unvoiced speech recognition in noise were analyzed through a 2 (group)  ×  2 (filtering)  ×  4 (masker) mixed-model ANOVA. There were significant main effects of group, *F*(1, 50)  =  6.584, *p*  =  0.013, *η_p_^2^*  =  0.116, of filtering, *F*(1, 50)  =  20.292, *p* < 0.001, *η_p_^2^*  =  0.289, and of masker, *F*(1, 50)  =  50.889, *p* < 0.001, *η_p_^2^*  =  0.504. There were significant interactions between group and masker, *F*(3, 150)  =  2.741, *p*  =  0.045, *η_p_^2^*  =  0.052, and between filtering and masker after Greenhouse-Geisser correction, *F*(2.545, 127.231)  =  5.851, *p*  =  0.002, *η_p_^2^*  =  0.105. There was no significant interaction between group and filtering or across the three factors.

**Figure 4.**
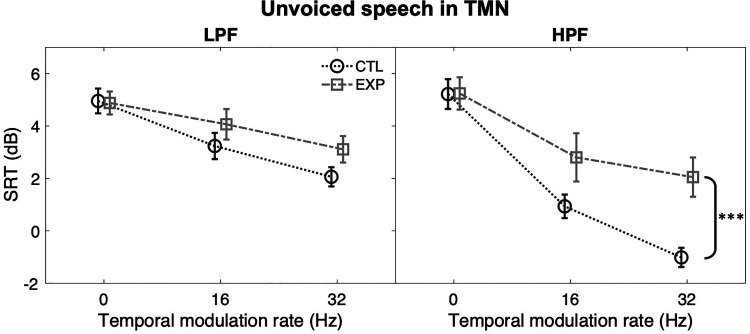
Difference between the CTL (dark grey) and the EXP (light grey) groups in SRTs of unvoiced speech in UN (0 Hz), 16-Hz and 32-Hz TMNs under LPF (left panel) and HPF (right panel) conditions. Error bars: SEM. *, *p* < 0.05; **, *p* < 0.01; ***, *p* < 0.001.

**Figure 5.**
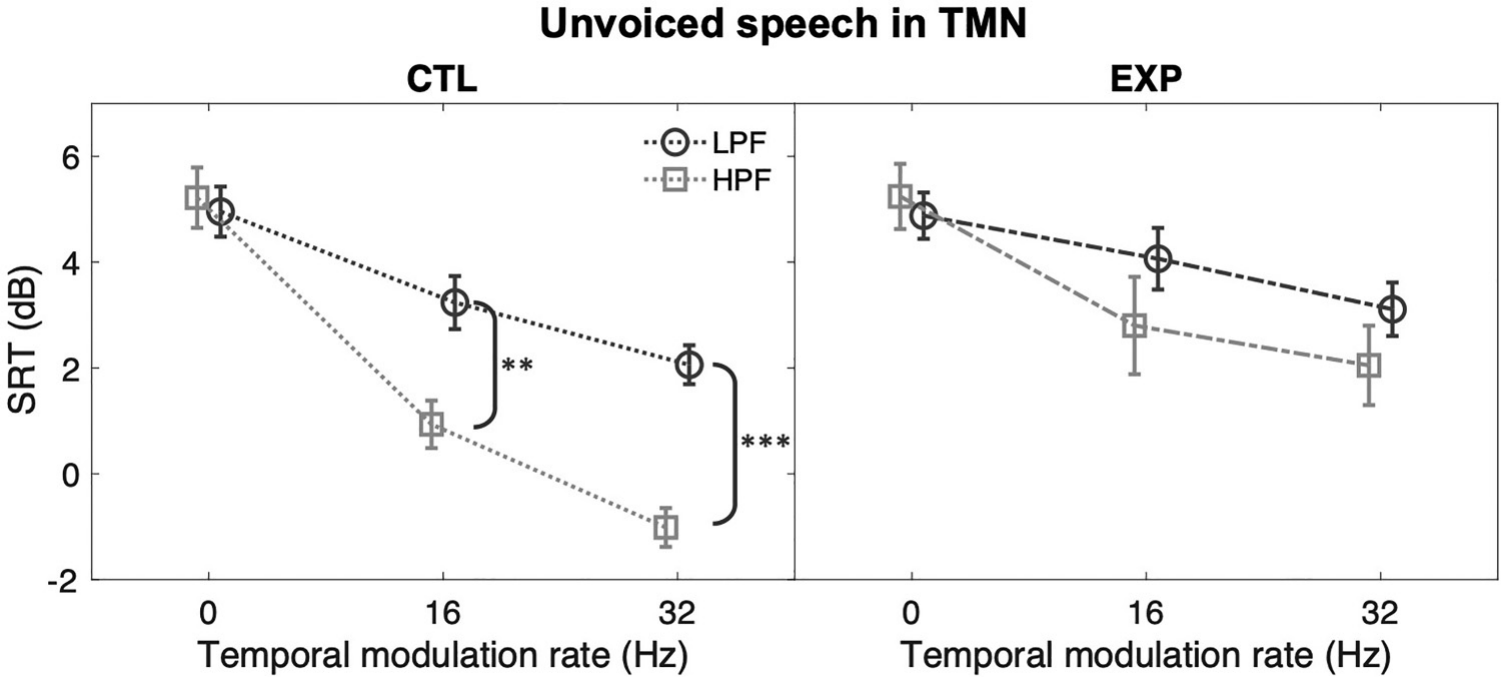
SRT differences between LPF (dark grey) and HPF (light grey) unvoiced speech in UN and TMNs within the CTL (left panel) and the EXP group (right panel). Error bars: SEM. *, *p* < 0.05; **, *p* < 0.01; ***, *p* < 0.001.

The effect of dental noise exposure on temporal envelope processing was examined by comparing the between-group performances when filtering and masker were controlled (Figure 4). All multiple comparisons used Bonferroni correction. The mean SRTs of the CTL group always appeared lower than those of the EXP group in all TMNs (LPF, 16-Hz: mean difference [MD]  =  0.8 dB; LPF, 32-Hz: MD  =  1.0 dB; HPF, 16-Hz: MD  =  1.9 dB; HPF, 32-Hz: MD  =  3.1 dB), but a statistically significant group difference was only observed for 32-Hz TMN, *F*(1, 50)  =  14.112, *p* < 0.001, *η_p_^2^*  =  0.220. Performance was also compared between the two filtering conditions within each group (Figure 5). When the modulation rate of the noise varied from 0 Hz (unmodulated) to 16 and 32 Hz, the between-filtering SRT differences increased for the CTL group, but not for the EXP group. The SRT differences for the CTL group were 2.3 dB for 16-Hz TMN, *F*(1, 50)  =  10.290, *p*  =  0.002, *η_p_^2^*  =  0.171, and 3.1 dB for 32-Hz TMN, *F*(1, 50)  =  19.351, *p* < 0.001, *η_p_^2^*  =  0.279. The SRT differences for the EXP group were about 1 dB (16-Hz: MD  =  1 dB; 32-Hz: MD  =  0.8 dB), *p* > 0.05.

**Figure 6.**
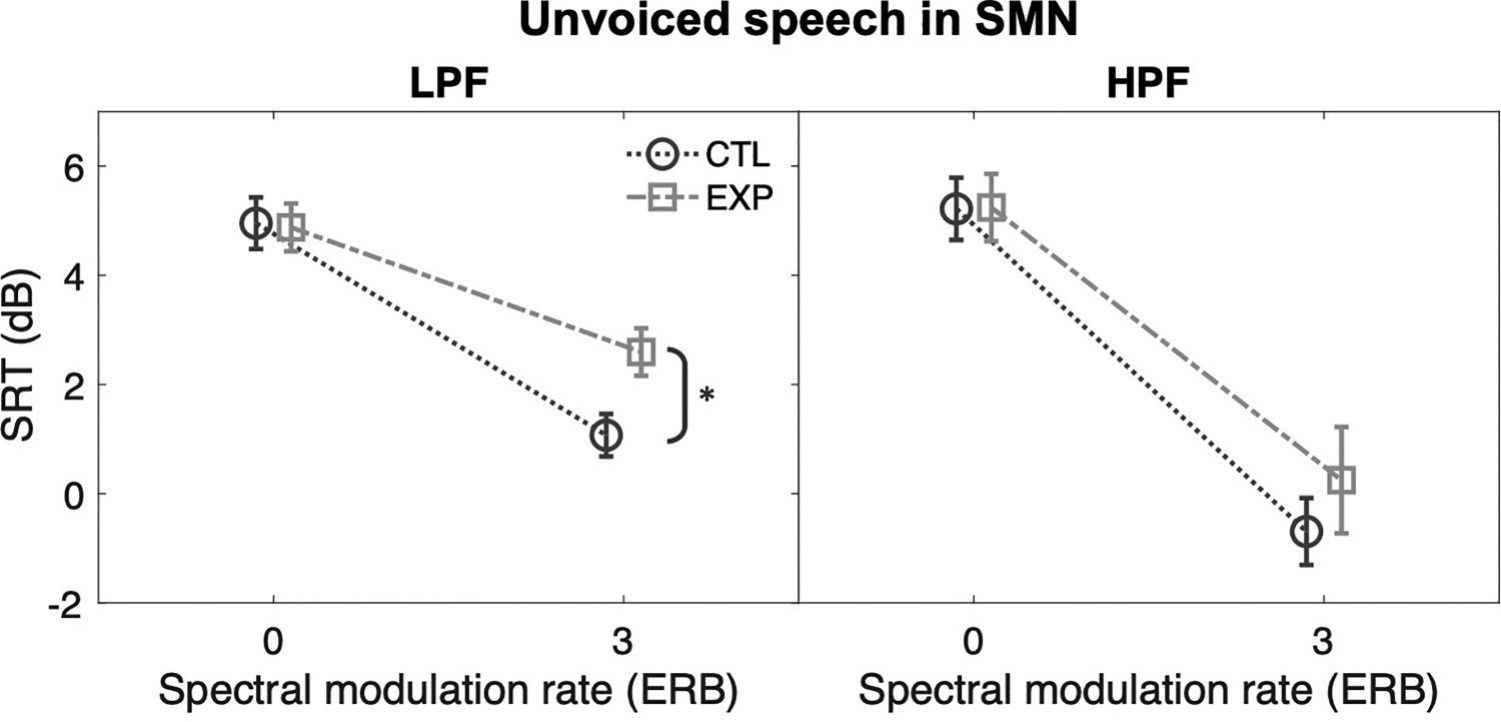
SRTs of unvoiced speech in SMN under LPF (left panel) and HPF (right panel) conditions. Error bars: SEM. *, *p* < 0.05; **, *p* < 0.01; ***, *p* < 0.001.

The effect of exposure on spectral envelope processing also was examined in the simple-effect analysis on performance in SMN (Figure 6). The mean SRTs of the CTL group appeared lower than those of the EXP group. The group difference was statistically significant only for LPF condition (MD  =  1.5 dB), *F*(1, 50)  =  6.853, *p*  =  0.012, *η_p_^2^*  =  0.121, but not for HPF condition (MD  =  0.9 dB), *p* > 0.05.

The SRTs for the two filtering conditions were also compared within the group. Both groups scored significantly lower SRTs for the HPF condition than for the LPF condition. The differences between the filtering conditions appeared larger for the EXP group than for the CTL group. The CTL group showed an SRT difference of 1.8 dB between the LPF and the HPF conditions, *F*(1, 50)  =  5.741, *p*  =  0.020, *η_p_^2^*  =  0.103. The EXP group an SRT difference of 2.3 dB, *F*(1, 50)  =  9.413, *p*  =  0.003, *η_p_^2^*  =  0.158.

### Contributions of Demographic and Audiological (Screening) Factors

Based on Figures 4 and 5, the EXP group performed more poorly on average than the CTL group in temporally or spectrally modulated noises, though the difference for the temporal condition did not reach statistical significance. It is of interest whether the participants’ demographic and audiological factors have contributed to the variations of the LPF unvoiced speech recognition. Therefore, LPF speech measures, including the SRTs of LPF unvoiced speech in 16-Hz TMN, in 32-Hz TMN, and in SMN, were analyzed using hierarchical multiple linear regression (HMLR). The basic demographic factors included age and years of musical training. The exposure-related demographic factors included the lifetime dental noise exposure L_eq_ (in dB SPL) and the lifetime non-dental noise exposure L_eq_ (in dB SPL). The variable for middle ear function was the average acoustic reflex amplitude (in ml) of low and middle frequencies (i.e. 0.5 to 2 kHz), which covered the frequency of the LPF unvoiced speech. The variables for inner ear outer hair cell (OHC) function were the average SNRs of DPOAE at low stimulus frequencies (0.552 to 2.783 kHz) and at high stimulus frequencies (3.506 to 8.838 kHz). The sequence of the factors adding into the HMLR analysis was 1) the basic demographic factors, 2) the exposure-related factors, 3) the variable of middle-ear function, and 4) the variable of inner ear OHC function. The EHF measures, such as EHF DPOAE and PTAs, were not correlated to the LPF speech measures. And as these measures examined the frequencies distant from the LPF speech frequencies (i.e. < 2.3 kHz), they were excluded from the regression analysis.

For LPF unvoiced speech in 16-Hz or 32-Hz TMNs, there were no significant correlations between the SRTs and any of the predictors, indicating no linear relationships between any of the demographic or audiologic factors and the SRTs of the LPF unvoiced speech in TMNs. Hence, linear regression was not conducted. For LPF unvoiced speech in SMN, the SRT was significantly correlated with lifetime dental noise exposure L_eq_, *r*  =  0.337, *p*  =  0.015. Tests of normality, homoscedasticity, and multicollinearity were not violated. The HMLR model was built, showing that lifetime dental noise exposure L_eq_ was the only significant predictor, while age, years of musical training, non-dental noise exposure, acoustic reflex amplitudes, or DPOAE amplitudes failed to account for the variance of the performance. The final model explained 19.9% of the variance in performance (Table 5).

**Table 5.** HMLR Model for the SRT of LPF Unvoiced Speech in SMN.

	Model 1			Model 2			Model 3			Model 4		
	B	SE	ß	B	SE	ß	B	SE	ß	B	SE	ß
Age	0.054	0.162	0.047	−0.088	0.158	−0.078	−0.088	0.16	−0.077	−0.092	0.172	−0.081
Years of music training	0.060	0.074	0.116	0.061	0.07	0.117	0.06	0.074	0.116	0.079	0.079	0.152
Dental L_eq_				0.022	0.008	0.368**	0.022	0.008	0.369*	0.021	0.008	0.366*
Non-dental L_eq_				−0.102	0.06	−0.23	−0.102	0.063	−0.231	−0.097	0.065	−0.221
Acoustic reflex amplitude							0.19	4.812	0.006	−0.374	5.166	−0.011
DPOAE amplitude (low frequency)										0.007	0.076	0.014
DPOAE amplitude (high frequency)										−0.068	0.078	−0.124
R^2^	0.017			0.185			0.185			0.199		
ΔR^2^	0.017			0.168			< 0.001			0.014		

Note. *, *p* < 0.05; **, *p* < 0.01.

## Discussion

The relationship between non-traumatic noise exposure and supra-threshold auditory envelope processing in human listeners was examined here. This study utilized unvoiced speech recognition that relies solely on temporal and spectral envelopes among dental-school students with quantifiable exposure to non-traumatic noise during professional training. A between-groups design was implemented to compare dental-school students to a cohort of peers not enrolled in dental school. The two groups showed no statistically significant differences in general demographic or audiological screening outcomes. Also, no difference was observed between-group on unvoiced speech recognition when the speech was presented in quiet or in unmodulated noise. When the noise was modulated, and listeners were required to exploit the temporal or spectral gaps with favorable SNRs to improve recognition, the group with dental noise exposure performed more poorly than their unexposed peers, and the poor use of spectral or temporal cues appeared dependent on stimulus frequency. Non-traumatic exposure to high-frequency dental noise was found to be associated with poor temporal envelope processing at higher frequencies but poor spectral envelope processing at lower frequencies. Given that the sound levels from the dental equipment typically do not exceed 80 dB SPL and students do not practice for more than 8 h per day, these results support the hypothesis that non-traumatic noise exposure may contribute to the degradation of supra-threshold envelope processing of speech that cannot be detected by routine audiological screenings.

The study first found that the experience of non-traumatic noise exposure could be related to poor temporal envelope processing. Listeners with dental noise exposure did not show as much masking release as the control listeners when recognizing the HPF unvoiced speech in TMN. When modulation rate was increased from 16 to 32 Hz, masking release increased in smaller magnitude for the EXP than for the CTL listeners. At higher modulation rates, the temporal gaps of the noise become briefer, so the task demands on temporal resolution for exploiting the information in the gaps could grow accordingly (Gustafsson & Arlinger, 1994; Dubno et al., 2003) and accentuate the poor temporal resolution of the compromised auditory system.

One of the plausible explanations for the links between noise exposure and poor supra-threshold temporal envelope processing is cochlear synaptopathy (Bharadwaj et al., 2014; Shaheen et al., 2015). Cochlear synaptopathy is a pathological change in the synapses of a selective group of auditory nerve fibers that can be induced by exposing the individual to noise that does not induce permanent threshold shift (Kujawa & Liberman, 2009; Lin et al., 2011; Furman et al., 2013). These auditory fibers normally encode the sound envelopes or intensity changes at high sound levels, varying the firing rates according to the input level changes while the firing rates of other fibers have saturated (Liberman, 1978). If these auditory fibers are damaged, one of the consequences is thought to be the degraded encoding of sound envelopes at supra-threshold levels. The current finding may also be explained by the reduced dynamic range adaptation in the neurons of the inferior colliculus (Bakay et al., 2018). Dynamic range adaptation refers to the ability of a neuron to shift its rate-level function toward the frequently occurring sound levels to avoid firing saturation and ensure high fidelity when encoding various sound levels (Dean et al., 2008). In Bakay et al. (2018), the amount of dynamic range adaption by the inferior colliculus neurons reduces after the noise exposure has induced cochlear synaptopathy, suggesting that the ability of temporal envelope coding could be impacted in the inferior colliculus in addition to cochlear synaptopathy.

The relation of temporal envelope processing and noise exposure has been examined previously, but the results have been conflicting. Kumar et al. (2012) found poorer AM detection thresholds for train drivers with normal audiograms than for age-matched unexposed individuals at 60 and 200 Hz modulation rates along with poor duration pattern test and speech reception in noise, supporting the temporal hypothesis of synaptopathy. Meanwhile, Paul et al. (2017, 2018) used AM detection with stimuli presented in narrow-band noise at various noise levels to limit off-frequency listening and to engage auditory fibers of different spontaneous rates. They did not consistently show poorer performance by young adults with higher noise exposure compared to their peers with lower exposure. Yeend et al. (2017) and Füllgrabe et al. (2020) controlled off-frequency listening by presenting the modulated targets in threshold-equalizing noise (TEN), which is configured to produce equal tone-in-noise thresholds for normal hearing listeners from 0.25 to 10 kHz and is used in the TEN test to diagnose cochlear dead regions (Moore et al., 2000), and they found no relationship between noise exposure and temporal processing. Stone et al. (2008) and Stone and Moore (2014) found worse AM discrimination for a noise-exposed group but only when the signals were presented near-threshold and not supra-threshold.

The lack of consistent previous evidence to associate temporal envelope processing and noise exposure suggest that conventional psychophysical measures of temporal envelope processing may not be sensitive enough or need further parameterization. This study has shown that unvoiced speech recognition in TMN can be an alternative and potentially more desirable measure to assess temporal envelope processing after non-traumatic noise exposure. Speech-based tasks provide the advantage of controlling off-frequency listening because speech intelligibility relies on the combined contributions of various frequency regions. The use of unvoiced speech can elicit high intelligibility in quiet^1^ despite the removal of pitch and harmonic information, which places greater emphasis on envelope cues. Then, bandpass filtering can constrain the examination to spectral regions of interest, such as those thought to be affected by noise exposure. The addition of TMN manipulates the test toward assessing temporal resolution and the efficiency of extracting information from the temporal gaps of modulated noise can be assessed using properly selected modulation rates, as has been shown in this study.

That said, when spectrally modulated noise was added, unvoiced speech recognition was weighed toward using spectral envelope cues. The current study also discovered that the experience of non-traumatic noise exposure could be related to spectral envelope processing. Listeners with dental noise exposure performed poorly when recognizing the LPF unvoiced speech in 3-ERB gapped SMN. The finding is not surprising. Without normal high-intensity auditory fibers, the rest of the fibers cannot fully represent the sound spectra at high intensities without firing-rate saturation, from which the reconstructed spectra may appear smoothed out with impoverished spectral details (Sachs & Young, 1979; May et al., 1996; Reiss et al., 2011). Furthermore, studies on the central auditory system have also reported negative impacts of non-traumatic noise exposure on neuronal spectral encoding, such as disrupted cortical tonotopic representation (Pienkowski & Eggermont, 2009, 2010a, 2010b; Pienkowski et al., 2011, 2013) and broadened neuronal frequency tuning curves (Zhou et al., 2011; Zhou & Merzenich, 2012; Kamal et al., 2013). Most of these studies have adopted moderate level of noise exposure (65 to 80 dB SPL) with prolonged exposure schedules (4 to 12 weeks), providing compelling evidence on the impact of prolonged non-traumatic noise exposure, such as dental noise, on spectral envelope processing.

It was interesting, however, to observe the performance difference between the two groups shifted from LPF to HPF speech when noise modulation switched from spectral into temporal domains. The exact reason behind the finding is yet unclear. One plausible explanation is that there is a trade-off between spectral and temporal resolution along the frequency axis and the degrees of spectral and temporal resolution vary from low to high frequencies. As the basilar membrane response is often modeled as a bank of Gammatone filters (Patterson et al., 1992; Lopez-Poveda & Meddis, 2001) and the impulse responses of the filters decreases in duration with increasing center frequencies, the temporal resolution of the auditory system may improve with increasing best frequencies while spectral resolution acts the opposite way. Another explanation is that the spetro-temporal trade-off along the frequency axis does not occur in the auditory system but in the importance weighting of speech cues. Previous studies using vocoded speech, which only relies on temporal envelope cues for speech recognition, have shown that despite temporal envelope cues appear important at all frequencies for speech in quiet, these cues are more heavily weighted at high frequencies than low frequencies for speech in noise (Apoux & Bacon, 2004; Ardoint et al., 2011; Fogerty, 2011).

It should be noted that the two groups did not appear different on the acoustic reflex amplitudes or the EHF measures, which were previously thought to be impacted by non-traumatic noise exposure (Valero et al., 2018; Liberman et al., 2016). Lack of reflex difference may be because that the acoustic reflex used in this study was a clinical screening, which had not controlled for spread of excitation at high stimulus intensities. Lack of the EHF threshold difference or DPOAE difference may be because of the different susceptibility to noise exposure in humans and animals. It will be interesting to examine whether group difference will emerge for the EHF measures on a long run, such as an early onset of EHF hearing loss in noise exposed group (Fernandez et al., 2015).

In conclusion, exposure to non-traumatic noise over time could be related to reduced envelope processing in humans in the absence of clinically defined audiological abnormalities. The finding supported the general hypothesis of cochlear synaptopathy, though central auditory dysfunction could also play a role. The effect of noise exposure could be related to both spectral and temporal processing and impact the two aspects of envelope processing in a frequency-dependent fashion. The task of recognizing unvoiced speech in modulated noise is shown to be usable in revealing supra-threshold envelope processing issues. However, the current study is limited in several aspects. A cross-sectional study coupled with a within-subject longitudinal design would be ideal for observing the noise exposure impact over time. It should be noted that despite the numbers of female and male participants were less balanced for the CTL than for the EXP groups, the result patterns remained when only female participants were examined and there was no significant performance difference between the males and the females within the EXP group (data not shown). Like all psychophysical studies, the current measure does not identify the site of lesions or the physiological mechanisms behind non-traumatic noise exposure. Approaches like computational modeling of pathological conditions in physiologically inspired auditory models may be useful to parameterize the current measure to differentially assess various site of lesions. Electrophysiological measures, such as envelope following responses, may also benefit the mechanistic study relating to temporal envelope processing and noise exposure. Overall, if the goal is to early discover the impact of noise exposure on hearing before measurable hearing threshold change, it is worth considering the inclusion of unvoiced speech recognition in a proposed test battery.

## Notes

1. Our pilot data (n = 5), young normal hearing listeners achieved near 100% correct performance for unfiltered unvoiced speech recognition.
